# Case Report: Ectopic CRH production by adrenal adenoma as a unique cause of the ACTH-dependent Cushing’s syndrome

**DOI:** 10.3389/fendo.2025.1649633

**Published:** 2025-08-25

**Authors:** Agata Pokrzywa, Janusz Pachucki, Małgorzata Bobrowicz, Łukasz Koperski, Urszula Ambroziak

**Affiliations:** ^1^ Department of Internal Medicine and Endocrinology, University Clinical Center of the Medical University of Warsaw, Warsaw, Poland; ^2^ Department of Internal Medicine and Endocrinology, Medical University of Warsaw, Warsaw, Poland; ^3^ Department of Pathology, Medical University of Warsaw, Warsaw, Poland

**Keywords:** Cushing’s syndrome, ectopic CRH secretion, adrenal adenoma, case report, ectopic Cushing’s syndrome

## Abstract

Isolated ectopic secretion of corticotropin-releasing hormone (CRH) is an exceedingly rare cause of Cushing’s syndrome (CS), accounting for fewer than 1% of cases. Ectopic CS is an uncommon but potentially life-threatening condition that often necessitates urgent diagnostic evaluation and treatment. Hormonal testing may suggest a pituitary origin, complicating the diagnostic process. To date, isolated ectopic CRH production has primarily been reported in cases of medullary thyroid carcinoma, pheochromocytoma, and various neuroendocrine tumors. We present the first documented case of adrenocorticotropic hormone (ACTH)-dependent CS resulting from CRH secretion by an adrenal cortical adenoma. A 68-year-old woman presented with severe hypercortisolemia and biochemically confirmed ACTH-dependent CS. Imaging revealed an unilateral adrenal mass without evidence of pituitary or extra-adrenal lesions. Dynamic endocrine testing was consistent with an ectopic source of ACTH. The patient underwent unilateral adrenalectomy, which led to full clinical and biochemical remission without prolonged adrenal insufficiency. Histopathological analysis confirmed an adrenal cortical adenoma showing focal immunoreactivity for CRH and absence of ACTH expression. This case highlights the importance of considering ectopic CRH secretion in the differential diagnosis of atypical ACTH-dependent CS, especially in patients presenting with adrenal adenomas.

## Introduction

Endogenous Cushing’s syndrome (CS) is the clinical manifestation of excessive cortisol secretion due to either adrenocorticotropic hormone (ACTH) – dependent or ACTH – independent mechanisms. ACTH-independent CS, caused mostly by adrenal cortical adenoma or carcinoma, is less common than the ACTH – dependent form, which accounts for approximately 80-85% of all CS cases (1–3). In the majority of ACTH – dependent cases, the source of ACTH is a corticotroph pituitary adenoma (Cushing’s disease). Less commonly, ectopic ACTH production is identified, occurring in approximately 10-20% of patients (4, 5). Tumors secreting corticotropin-releasing hormone (CRH) are exceedingly rare and are often associated with co-secretion of ACTH (1). Ectopic CRH secretion is estimated to account for fewer than 1% of all CS cases (3, 6).

Isolated ectopic CRH production has primarily been described in cases of medullary thyroid carcinoma and pheochromocytoma. A few additional cases with alternative etiologies—most notably neuroendocrine neoplasms—have also been described (7, 8). However, to our knowledge, the ectopic CRH production by an adrenal adenoma has not previously been reported in the literature.

Ectopic CS is a rare but frequently severe disease. Due to the pronounced hypercostisolemia, patients often present with an endocrine emergency requiring urgent evaluation and management (1). Prompt and accurate diagnostic workup – including various hormonal testing and advanced imaging – is essential and should be initiated together with both preventive and therapeutic management of comorbidities and complications to delay rapid clinical deterioration or fatal outcomes.

Ectopic production of CRH and/or ACTH may be associated with diagnostic difficulties – suggesting a pituitary source of excessive ACTH secretion in hormonal testing. Neither single diagnostic test nor combination of tests can definitively distinguish between pituitary and ectopic ACTH secretion; discordant results occur in up to one-third of cases (9). Current recommendations rely on combination of dynamic testing, especially CRH and desmopressin stimulation, high dose dexamethasone suppression test (HDDST) plus pituitary MRI, followed by whole – body Computed Tomography (CT) if results are equivocal (9). Bilateral inferior petrosal sinus sampling (IPSS), which has long been the gold standard to reliably exclude ectopic ACTH production, may yield both false-negative and false-positive results in ectopic CS (9).

We present a unique case of CS due to ectopic CRH production from an adrenal adenoma.

## Case description

### Initial diagnostic evaluation in the internal medicine department

A 68 – year – old woman with a history of long – term hypertension and type 2 diabetes mellitus was admitted to a local hospital due to weakness and severe lower extremity edema. Laboratory investigations revealed marked hyperglycemia (741 mg per deciliter) and profound hypokalemia (1.77 mmol per liter; normal range, 3.50 to 5.00 mmol per liter). Initial hormonal evaluation confirmed ACTH – dependent hypercortisolemia with morning cortisol level of 1185 nmol per liter (normal range, 166 to 507 nmol per liter) and evening cortisol (2300 h) of 771.4 nmol per liter (normal range, 73.8 to 291 nmol per liter), in the presence of high normal ACTH level (44.97 pg per milliliter [normal range, 10.0 to 60.0 pg per milliliter]).

**Figure 1:**
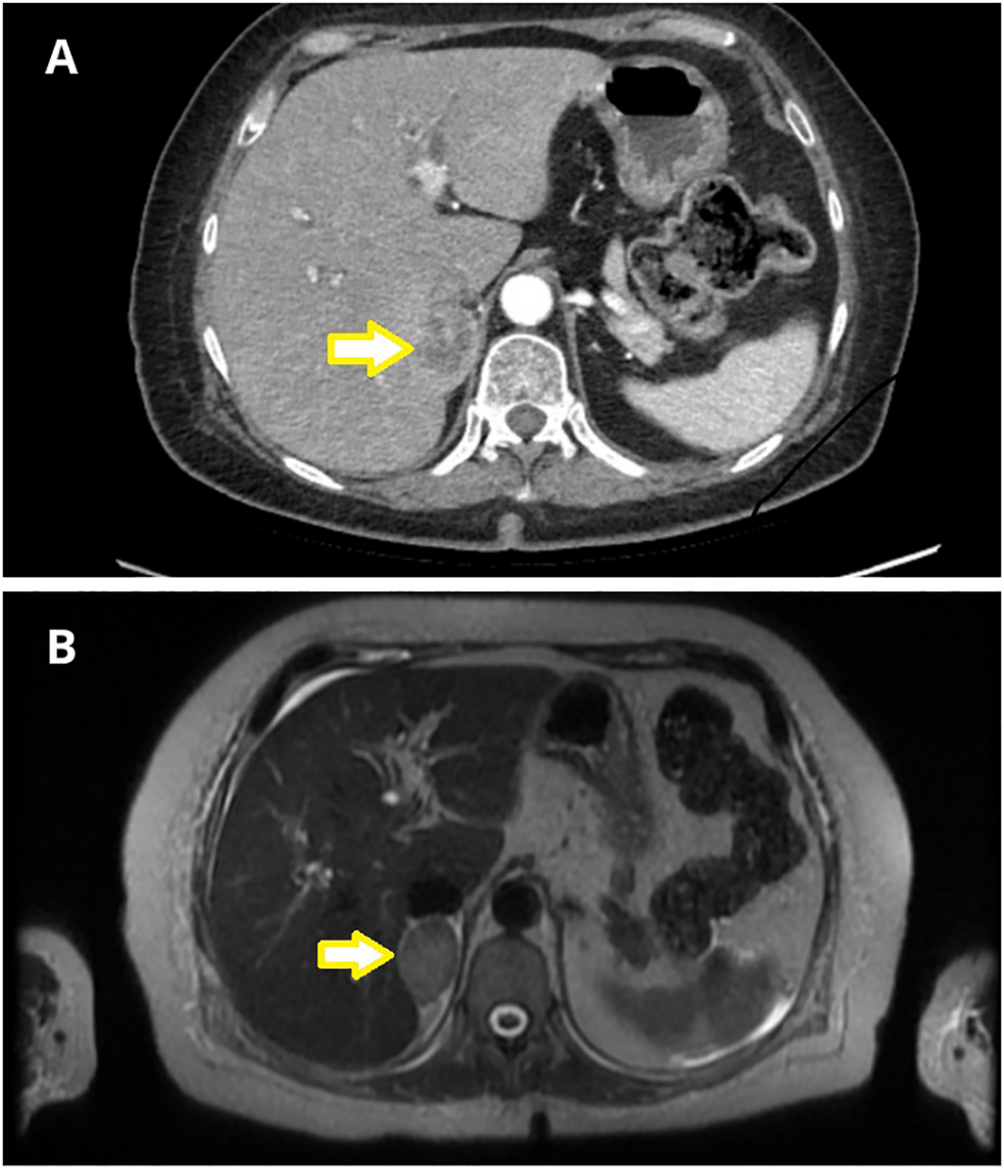
Adrenal imaging with nodular lesion marked with an arrow. **(A)** Abdominal Computed Tomography (CT) revealed a nodular lesion in the right adrenal gland, measuring 38x24 mm, extending into the liver parenchyma. **(B)** Abdominal Magnetic Resonance Imaging (MRI) demonstrated a focal lesion in the upper pole of the right adrenal gland, measuring 34x23x29 mm (T2 sequence).

Abdominal CT identified a round lesion in the right adrenal gland measuring 38 × 24 mm, extending into the liver parenchyma (**Figure 1A**). The average lesion density was 35 HU in the native phase and 91 HU in the venous phase – suggesting the possibility of malignant neoplasm. Subsequent abdominal magnetic resonance imaging (MRI) revealed a 34 × 23 × 29 mm round lesion in the upper pole of the right adrenal gland, with peripheral contrast enhancement at one minute and no diffusion restriction (**Figure 1B**). Both CT and MRI revealed no abnormalities in the left adrenal gland.

**Figure 2:**
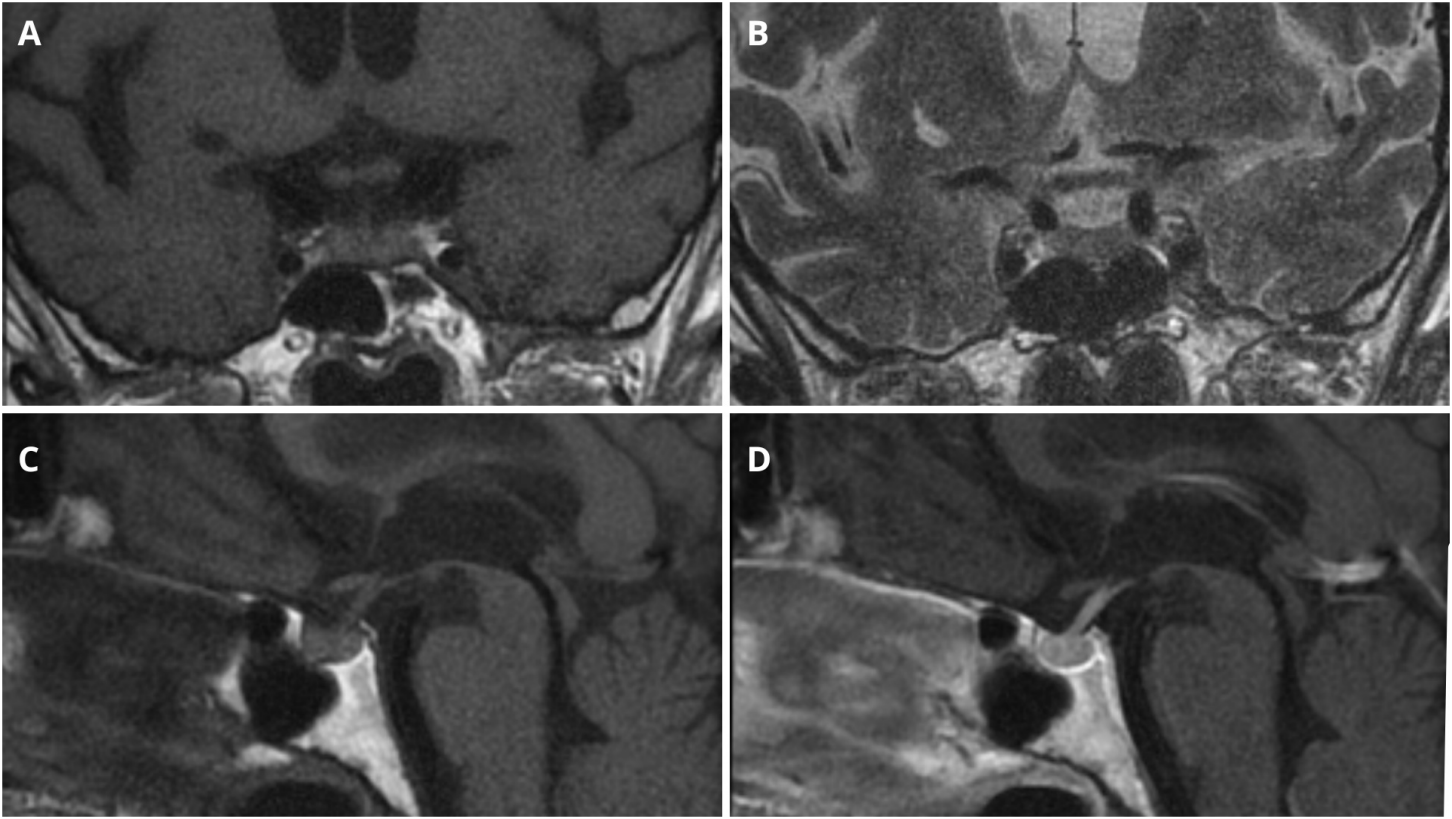
Pituitary MRI imaging. **(A)** Coronal T1-weighted sequence. **(B)** Coronal T2-weighted sequence. **(C)** Sagittal T1-weighted sequence before intravenous contrast administration. **(D)** Sagittal T1-weighted sequence after intravenous contrast administration.

Pituitary MRI demonstrated a structurally normal gland, measuring 6 × 9 × 14 mm, without focal abnormalities (**Figure 2**).

The clinical course was complicated by urosepsis due to Escherichia coli, pneumonia, esophageal candidiasis and anemia requiring several red blood cells transfusions.

The patient was transferred to the endocrinology unit for further evaluation and treatment. By the time of the referral, she exhibited severe Cushingoid features, including central obesity, round face, facial plethora, hirsutism, supraclavicular and dorsocervical fat pads, thin skin, and pronounced lower extremities edema. The intensity of her hypercortisolism was reflected in numerous complications, including uncontrolled diabetes, hypertension, severe hypokalemia, and respiratory failure secondary to influenza A infection with bacterial superinfection.

### Endocrinological assessment

**Figure 3:**
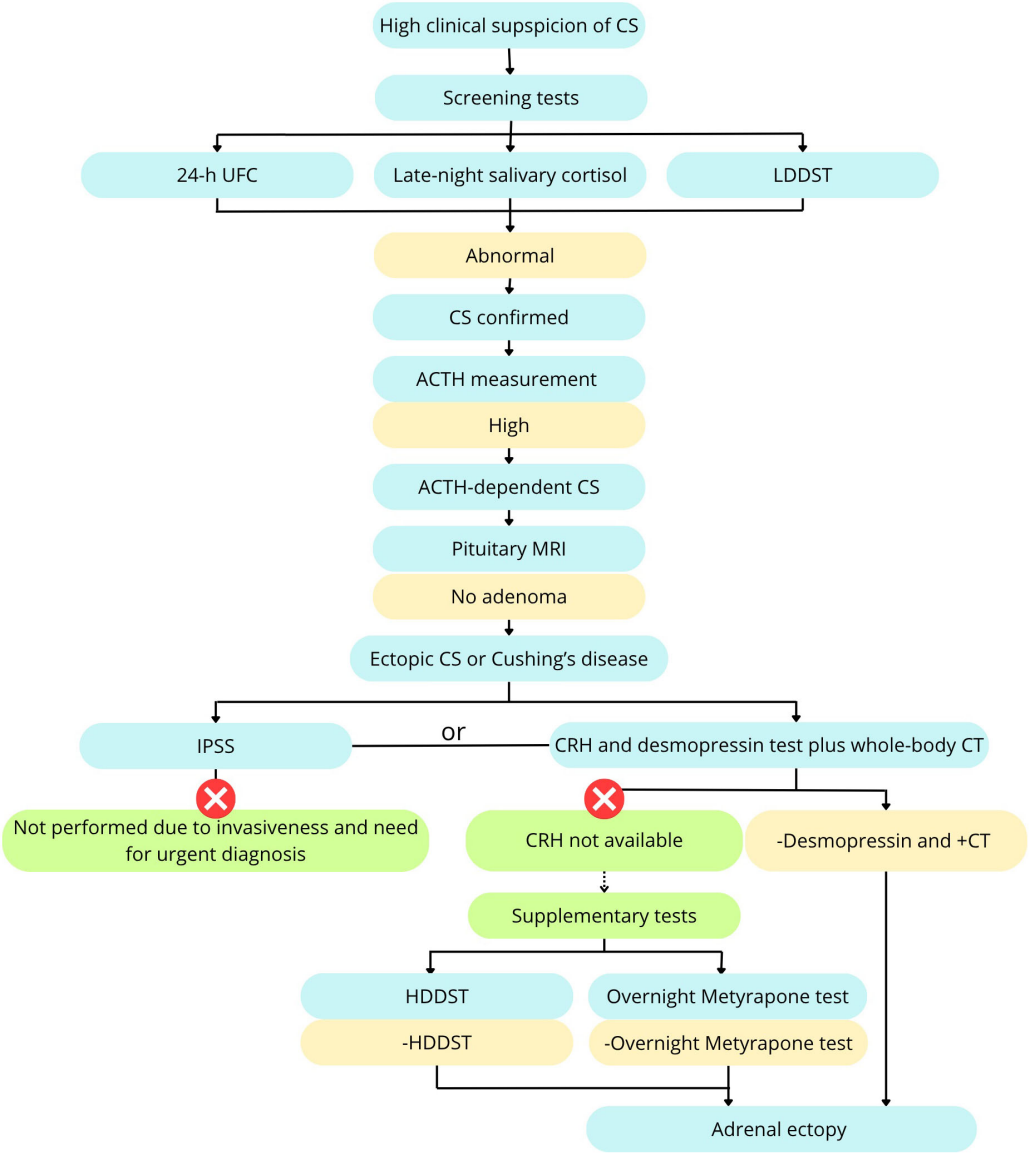
Diagnostic algorithm including supplementary tests. Based on Fleseriu et al. (9), modified.

As part of the endocrinological assessment, we conducted several dynamic tests. The diagnostic algorithm is presented in **Figure 3**. At the beginning we performed several screening tests – 24 – hour Urine Free Cortisol (UFC), late night salivary cortisol and the low-dose dexamethasone suppression test (LDDST). LDDST was performed over 48-hour period, involving oral dexamethasone (DXT) administration at a dose of 0.5 mg every 6 hours. Baseline and post-test serum cortisol concentrations, as well as 24-hour UFC levels, were measured. Reference cutoffs were serum cortisol <1.8 ug/dL and ≥50% suppression of UFC (10).

Subsequently, a HDDST was conducted, using oral DXT administration at a dose of 2 mg every 6 hours for 48 hours. The predefined threshold was ≥50% suppression of serum cortisol and UFC (2).

An overnight metyrapone test was conducted by oral administration of 1500 mg of metyrapone at 2200 h and measurement of morning serum cortisol and plasma ACTH. Given the lack of fully validated diagnostic criteria for CS, we employed the metyrapone test as a supplementary investigation. Findings consistent with an ectopic source of ACTH included a significant cortisol suppression and an insignificant rise in ACTH levels.

A desmopressin stimulation test (desamino-8-D-arginine vasopressin – DDAVP) stimulation test was conducted with measurements of plasma ACTH and serum cortisol obtained at −10 minutes, just before and at 10, 15, 30, and 45 minutes following the intravenous administration of 8 µg of 1-desamino-8-D-arginine vasopressin. A positive response was defined as ≥50% increase in ACTH or ≥20% increase in cortisol (2).

The results of dynamic testing indicated an ectopic ACTH secretion are summarized in **Table 1**. Neither LDDST nor HDDST demonstrated suppression of serum cortisol levels or UFC excretion. The metyrapone stimulation test revealed a marked reduction in serum cortisol concentration with no ACTH stimulation. During the desmopressin stimulation test (DDAVP), no increase in either serum cortisol or plasma ACTH levels was observed.

**Table 1:** Laboratory presentation.

ENDOCRINE WORK-UP	Value	Reference Range
Morning Plasma ACTH [pg/ml]	123	7.20 – 63.3
Evening Plasma ACTH [pg/ml]	109	7.20 – 63.3
Morning Serum Cortisol [µg/dl]	61.5	4.82 – 19.5
Evening Serum Cortisol [µg/dl]	59.7	2.47 – 11.9
Evening Salivary Cortisol [µg/dl]	25.3	<0.4
24 hour Urine Free Cortisol [µg/24h]		4.00 – 176
Sample 1	3670	
Sample 2	3087	
Low Dose Dexamethasone Suppression Test		
Morning Serum Cortisol [µg/dl]	69.0	< 1.80 µg/dl
24 hour Urine Free Cortisol [µg/24h]	3458	≥ 50% decrease
High Dose Dexamethasone Suppression Test		
Morning Serum Cortisol [µg/dl]	81.2	≥ 50% decrease
24 hour Urine Free Cortisol [µg/24h]	7142	≥ 50% decrease
Overnight Metyrapone Test		
Morning Serum Cortisol [µg/dl]	8.39	decrease ^a^
Morning Plasma ACTH [pg/ml]	222	increase ^a^
Desmopressin Stimulation Test		
Serum Cortisol [µg/dl]		≥ 20% increase
-10 min	12.4	
0 min	12.2	
+10 min	12.1	
+15 min	12.2	
+30 min	11.6	
+45 min	11.0	
Plasma ACTH [pg/ml]		≥ 50% increase
-10 min	208	
0 min	180	
+10 min	159	
+15 min	148	
+30 min	132	
+45 min	133	

Evening hormone measurements were performed at 2300 h.

ACTH, adrenocorticotropic hormone.

a specific cut-off points are not defined.

Plasma fractionated metanephrine levels remained within the normal reference range.

Chest CT revealed no lesions suggestive of any ectopic ACTH source, and the adrenal mass was considered the most likely origin of ectopic ACTH production.

### Treatment

Due to the severity of the condition, the patient required immediate treatment, particularly for hypokalemia and hyperglycemia. Significant lower extremity edema limited the use of intravenous therapy, necessitating primarily oral treatment. Initially, the patient received 40 mmol of intravenous potassium chloride and 60 mmol orally, under continuous cardiac monitoring. During hospitalization, oral potassium chloride supplementation was gradually increased to a maximum of 160 mmol per day. No gastrointestinal side effects were observed. In addition, the patient was treated with oral spironolactone at a daily dose of 400 mg to manage both edema and hypokalemia. For hyperglycemia, subcutaneous insulin therapy was initiated, including rapid-acting insulin aspart (up to 46 units/day) and long-acting insulin glargine (up to 32 units/day administered in two divided doses).

Right after dynamic testing, initial treatment of 750 mg of metyrapone and 400 µg of octreotide daily was conducted, followed by intramuscular injection of 120 mg of lanreotide.

Following clinical stabilization and achievement of preoperative targets (fasting serum glucose <200 mg/dL, serum potassium >3.5 mmol/L, and morning serum cortisol of 12.1 µg/dL), the patient underwent right-sided laparoscopic adrenalectomy. On the day of surgery, the patient received hydrocortisone supplementation at a dose of 100 mg every 12 hours.

### Histopathological examination

Histopathological examination included routine hematoxylin and eosin (H&E) staining, along with immunohistochemical (IHC) analysis targeting chromogranin A (CgA), CRH, and ACTH. ACTH was stained using the ACTH Polyclonal antibody (rabbit, Roche, Basel, Switzerland) and CRH using the CRH/CRF Polyclonal antibody (rabbit, Proteintech, Rosemont, USA, Cat# 10944-1-AP). The Ki-67 proliferation index was assessed on an immunohistochemically stained slide scanned using a Hamamatsu NanoZoomer 2.0-HT scanner and analyzed with NDP.view2 software. Initially, low magnification was used to identify regions with the highest density of immunopositive cells (hotspots). Quantification was then performed at medium magnification (20×), counting at least 1,000 cells across three hotspots. The final result was expressed as the percentage of Ki-67–positive cells among all counted cells.

**Figure 4:**
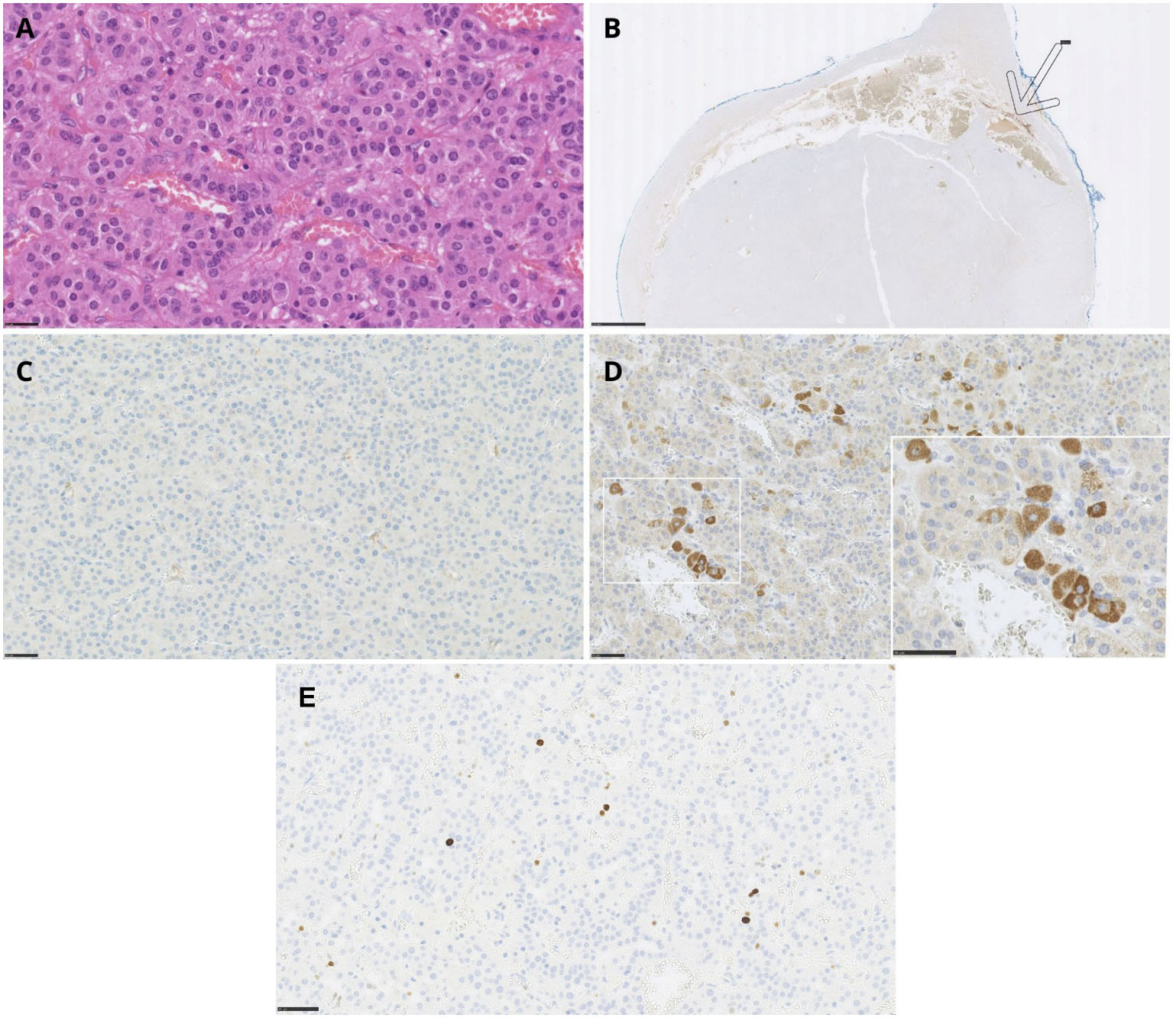
Adrenal cortical adenoma – histopathology and immunohistochemical staining of the tumor. **(A)** Adrenal cortical adenoma, H&E staining (scale bar: 25 μm). **(B)** Negative chromogranin A immunostaining in the tumor with positive reaction in adrenal medulla - shown by an arrow (scale bar: 2.5 mm). **(C)** Negative ACTH immunostaining (scale bar: 50 μm). **(D)** Positive expression of CRH in tumor cells (scale bar: 25 μm, insert scale bar 50 μm). **(E)** The Ki-67 immunostaining in adrenal cortical adenoma (scale bar: 50 μm).

Macroscopic examination of the excised adrenal gland revealed a well-circumscribed, firm, and unencapsulated tumor measuring 3.1 × 2.7 × 2.4 cm. In contrast to preoperative imaging findings, there was no evidence of infiltration into surrounding adipose tissue or adjacent organs. Histopathological analysis confirmed the diagnosis of an adrenal cortical adenoma, without features suggesting malignancy. Specifically, there was no significant nuclear pleomorphism, atypical mitotic activity, necrosis, or evidence of capsular, vascular, or sinusoidal invasion. The Ki-67 proliferation index was low, at 1–2% (**Figure 4E**).

Routine H&E staining is shown in **Figure 4A**. IHC analysis revealed no immunoreactivity for chromogranin A or ACTH (**Figures 4B, C**). However, a positive staining was observed for CRH (**Figure 4D**), confirming ectopic CRH production by the tumor.

### Postoperative course

Following surgery, the patient achieved complete clinical and biochemical remission of CS, with unexpectedly rapid recovery of the hypothalamic–pituitary–adrenal axis after few days and no prolonged secondary adrenal insufficiency. On the day of surgery and the following day, the patient received intravenous hydrocortisone at a dose of 100 mg every 12 hours. Over the next two days, oral hydrocortisone was administered at doses of 20 mg in the morning, 20 mg at midday, and 10 mg in the evening (8:00 AM, 1:00 PM, and 6:00 PM). On postoperative days 3 and 4, the dosage was tapered to 20–10–10 mg based on the clinical assessment. On postoperative day 5, the morning serum cortisol level was 15.6 µg/dL, prompting the discontinuation of hydrocortisone. The following day, the morning cortisol level was 20.8 µg/dL, with a preserved circadian rhythm; the evening cortisol was 6.36 µg/dL.

Trend of biochemical parameters at the initial visit, and during preoperative and postoperative periods is presented in **Table 2**.

**Table 2:** Trend of biochemical parameters during hospitalization in the Department of Endocrinology.

Parameter	Initial visit	Preoperatively	Postoperatively	Reference ranges
Fasting Plasma Glucose [mg/dl]	307	198	176	70.0 – 99.0
Serum Potassium [mmol/l]	2.35	4.14	4.23	3.60 – 5.10
Serum Sodium [mmol/l]	142.4	143	142	135 – 145
Morning Serum Cortisol [µg/dl]	61.5	12.1	20.8	4.82 – 19.5
Evening Serum Cortisol [µg/dl]	59.7	ND	6.36	2.47 – 11.9
Morning Plasma ACTH [pg/ml]	123	62.6	45.1	7.20 – 63.3
Evening Plasma ACTH [pg/ml]	109	ND	41.4	7.20 – 63.3
24 hour Urine Free Cortisol [µg/24h]	3670	7142	29	4.00 – 176
Evening Salivary Cortisol [µg/dl]	25.3	ND	0.09	<0.4

ACTH, adrenocorticotropic hormone; ND, Not Determined.

**Figure 5:**
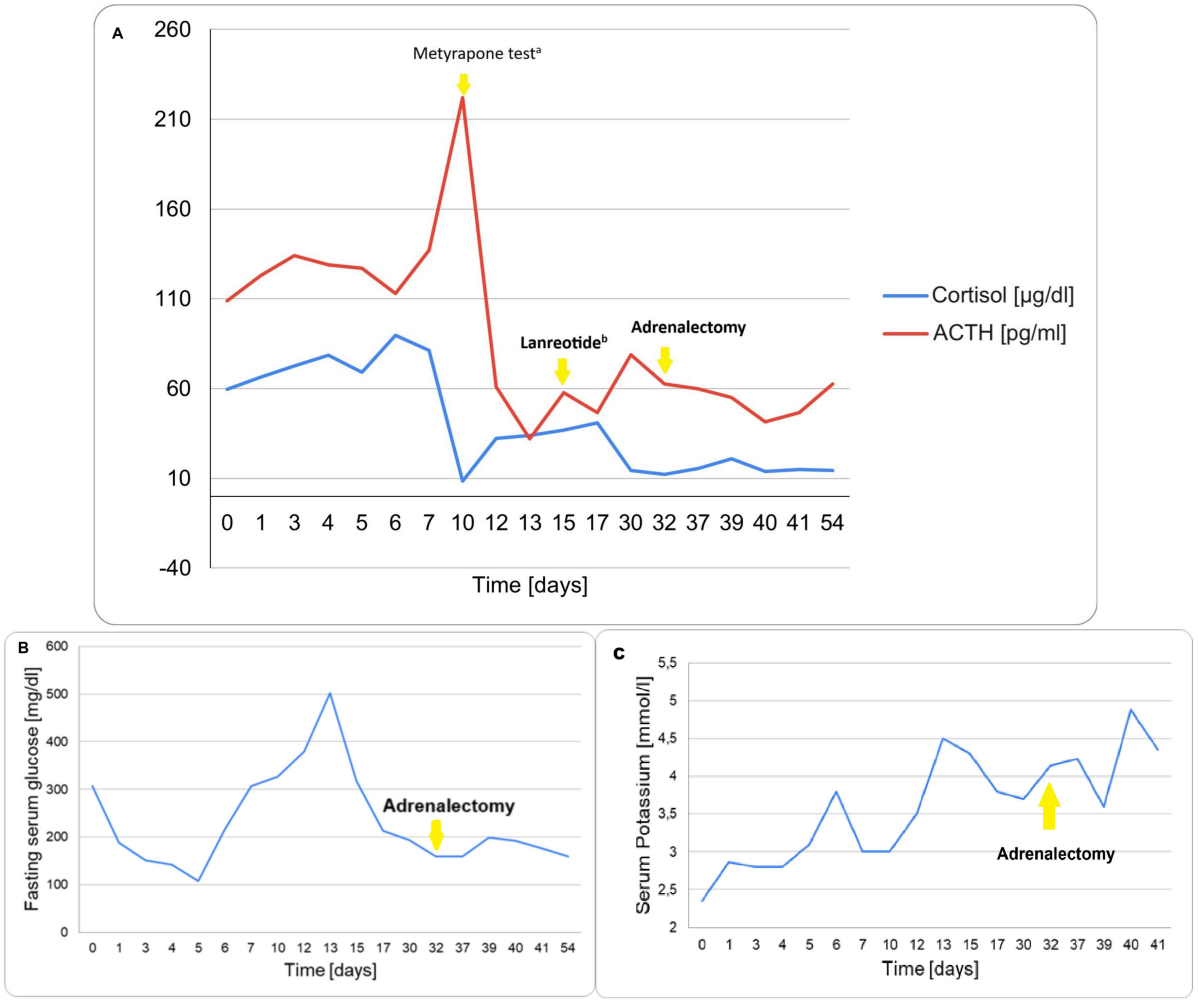
Trend of key biochemical parameters during the clinical course. **(A)** Morning Cortisol and Plasma ACTH levels. ^a^Metyrapone overnight test (1500 mg), followed by therapy of 750 mg of metyrapone and 400 µg of octreotide daily. ^b^Intramuscular injection of 120 mg of lanreotide. **(B)** Fasting serum glucose. **(C)** Serum Potassium.

Selected parameters are presented graphically in **Figure 5**, including morning cortisol and ACTH concentrations (**Figure 5A**), fasting serum glucose (**Figure 5B**), and serum potassium levels (**Figure 5C**).

A postoperative PET [68Ga]Ga-DOTA-TATE scan revealed no somatostatin receptor – expressing lesions.

## Discussion

We present a unique case of CS resulting from ectopic CRH secretion from an adrenal adenoma. To the best of our knowledge, this form of ectopic CRH production has not been previously reported in the literature.

Ectopic CRH production is accounting for less than 1% of CS cases (3, 6). Nakhjavani et al., in their review from 1971 to 2018 found only 31 patients with pure CRH secretion, confirmed by immonuhistochemical staining positive for CRH and negative for ACTH (7). In the years that followed, only a few additional cases of isolated ectopic CRH production have been reported to date (3, 11–13). The most commonly reported tumor types include pheochromocytoma and medullary thyroid carcinoma, followed by bronchial carcinoids, thymic carcinoids, pancreatic neuroendocrine tumors, and other less frequently observed neoplasms. CRH secretion from ectopic sources can stimulate ACTH secretion via both local autocrine and systemic endocrine mechanisms, exacerbating hypercortisolemia (14). Patients with ectopic CRH secretion tend to present with more severe and rapidly progressive disease than those with Cushing’s disease, with a higher risk of infectious and metabolic complications (1, 15).

Our patient presented with severe hypercortisolemia. The clinical urgency of her condition necessitated rapid initiation of steroidogenesis inhibition with metyrapone and a somatostatin analogue. Adrenalectomy led to almost immediate complete remission, consistent with reports that ectopic CRH-related CS may resolve more quickly than ectopic ACTH-dependent cases (16). The underlying pathophysiological mechanism is likely related to the direct stimulatory effect of CRH on pituitary corticotrophs, which may allow for a more rapid restoration of endogenous ACTH secretion following tumor resection. In contrast, ectopic ACTH-producing tumors can lead to prolonged corticotroph suppression, often resulting in more prolonged and severe secondary adrenal insufficiency.

Differential diagnosis of ACTH-dependent CS remains a considerable clinical challenge. It is well established that ectopic ACTH secretion may occasionally respond to DXT suppression testing or be stimulated by CRH or DDAVP, thereby mimicking the biochemical profile of Cushing’s disease (9, 17, 18).

The presence of ectopic CRH secretion further complicates interpretation of dynamic endocrine tests. CRH – driven stimulation of ACTH production may result in false-positive results during central venous sampling (3, 14, 19). Since ACTH and cortisol secretion in these cases is not autonomous but rather driven by CRH excess, a HDDST may produce apparent cortisol suppression. Such misleading results, including HDDST suppression combined with a positive CRH stimulation test, have been previously described by Young et al. (8).

Imaging interpretation also poses challenges. The small size of many pituitary microadenomas, as well as ectopic ACTH-secreting tumors, can lead to false-negative imaging results. Conversely, pituitary lesions identified on MRI may represent incidental non-functioning adenomas (9, 13). Accordingly, current guidelines recommend correlating clinical findings with the results of at least three or four diagnostic tests, typically including pituitary MRI, CRH and DDAVP stimulation tests, and whole-body CT in cases with inconclusive results (9). HDDST or metyrapone ACTH stimulation may also be included as an alternative dynamic test.

In our case, the diagnostic process included extended LDDST, HDDST, DDAVP stimulation test, pituitary MRI, and chest and abdominal CT. Due to the unavailability of CRH, a DDAVP and metyrapone test was performed to supplement the evaluation. Although no fully validated diagnostic criteria exist for interpreting metyrapone testing in CS, a significant cortisol suppression and insignificant increase in ACTH levels was suggestive of an ectopic source (20). The concordant results of HDDST, DDAVP, and metyrapone testing pointed toward ectopic ACTH secretion.

The diagnosis of ectopic CRH production relies on IHC staining. Therefore, if no CRH-specific staining is performed, the diagnosis may be easily missed (19). In our case, imaging revealed only a right adrenal mass, with no other suspicious lesions. Given its imaging characteristics and clinical presentation, the adrenal tumor was initially suspected to be the source of ectopic ACTH production. However, IHC analysis of the resected tumor revealed negative staining for ACTH and positive immunoreactivity for CRH, thereby confirming ectopic CRH secretion. The pathology result was consistent with the clinical observation of lack of secondary adrenal insufficiency after surgery, what spoke for ectopic CRH production.

## Conclusions

Isolated ectopic secretion of CRH is an extremely rare cause of Cushing’s syndrome, but its potentially life-threatening course necessitates urgent diagnostic evaluation and treatment. To our knowledge, this is the first reported case of a CRH-producing adrenocortical adenoma. We believe that this case may help raise awareness of either ectopic ACTH or CRH syndromes in patients with ACTH-dependent CS, particularly in the context of adrenal adenomas.

## Data Availability

The original contributions presented in the study are included in the article/supplementary material. Further inquiries can be directed to the corresponding author.
